# Efficient thermoelectric energy conversion on quasi-localized electron states in diameter modulated nanowires

**DOI:** 10.1186/1556-276X-6-286

**Published:** 2011-04-04

**Authors:** Xanthippi Zianni

**Affiliations:** 1 Department of Applied Sciences, Technological Institution of Chalkida, Psachna, 34400 Evia, Greece

## Abstract

It is known that the thermoelectric efficiency of nanowires increases when their diameter decreases. Recently, we proposed that increase of the thermoelectric efficiency could be achieved by modulating the diameter of the nanowires. We showed that the electron thermoelectric properties depend strongly on the geometry of the diameter modulation. Moreover, it has been shown by another group that the phonon conductivity decreases in nanowires when they are modulated by dots. Here, the thermoelectric efficiency of diameter modulated nanowires is estimated, in the ballistic regime, by taking into account the electron and phonon transmission properties. It is demonstrated that quasi-localized states can be formed that are prosperous for efficient thermoelectric energy conversion.

## Introduction

A measure of the thermoelectric efficiency of a material is the dimensionless figure of merit *ZT *≡ *S*^2^*σT*/*κ*, where σ is the conductivity, *S *is the thermopower, *κ *is the thermal conductivity and *T *is the absolute temperature. In nanostructures, quantum confinement of electrons and phonons favours their thermoelectric transport properties, resulting in increased thermoelectric efficiency [1]. Nanowires and arrays of nanodots are currently attracting much research interest. It has been theoretically shown that nanodots can have very high thermoelectric efficiencies due to their discrete energy spectrum [2-4]. Quantum confinement causes enhancement of *ZT *in nanowires. Considerably high values of the figure of merit have been found in very thin wires [5-8]. Despite the noticeable progress in the fabrication of wires with high aspect ratios, the poor mechanical properties of very thin wires is a drawback for developing devices based on them.

Recently, we proposed that increase of the thermoelectric efficiency could be achieved by modulating the diameter of the nanowires [9]. We showed that the electron thermoelectric properties depend strongly on the geometry of the diameter modulation. In this Letter, we focus on the electron propagation states that we reported to have high values of thermoelectric figure of merit when phonon conduction was neglected. The thermoelectric efficiency of these states is estimated, here, by taking into account the electron and phonon transmission properties. In what follows, it is shown that efficient thermoelectric energy conversion can be achieved on electron quasi-localized states in diameter modulated nanowires.

### Theoretical model

The relation between the thermoelectric transport coefficients and the transmission coefficient *T*(*E*) is given by Landauer-Büttiker formalism:(1)(2)(3)(4)

where *G *is the conductance, *S *is the thermopower, *K *is the thermal coefficient and *κ*_e _is the electron thermal conductance. The symbol *f *denotes the Fermi distribution function and *E*_F _is the Fermi energy. The thermoelectric efficiency is measured by the dimensionless figure of merit:(5)

where *κ*_ph _is the phonon thermal conductance. An optimal thermoelectric efficiency *ZT*_0 _can be estimated considering only the electronic contribution in the thermal conductance, whereby(6)(7)(8)

The coefficient *α *is a measure of the effect of phonon conduction on the thermoelectric efficiency. When phonon conduction is non-negligible, the coefficient *α *is smaller than 1 and *ZT *is smaller than *ZT*_0_.

## Results and discussion

**Figure 1:**
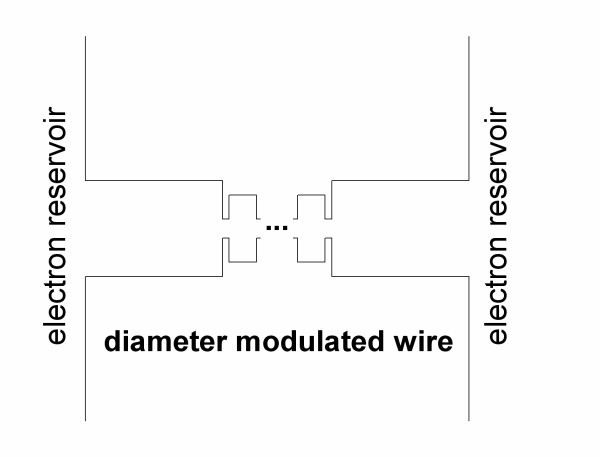
**Sketch diagram of the diameter modulated nanowire**. Two-dimensional GaAs wires have been used for illustration. The dimensions of the diameter modulations are: (a) the thick parts are 100 nm long and 50 nm thick, (b) the thin constrictions are 5 nm long and 10 nm thick, and (c) the dots are 20 nm long and 35 nm thick.

**Figure 2:**
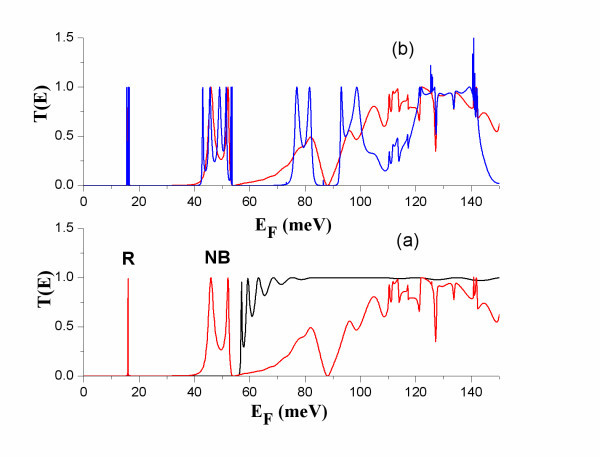
**The energy dependence of the transmission coefficient**. *T*(*E*) is shown for: **(a) **a uniform straight wire 10 nm thick (black) and of a wire modulated by one dot (red), **(b) **a wire modulated by one dot (red) and by three dots (blue).

The formalism of the previous section explicitly shows that the transport properties of an electron propagating through a wire are sensitive to the energy dependence of the transmission coefficient. Energy selectivity is provided for electrons by their Fermi distribution at the electrodes. The electron Fermi energy, *E*_F_, varies depending on the electrode material and/or doping. It can also be varied electrostatically by an external gate in a gated-wire configuration. The transmission coefficient of an electron travelling ballistically through a uniform straight wire is a step-like function of its Fermi energy, *E*_F _[10]. If we now consider a wire with diameter modulation by units that assume discrete energy spectra, e.g. quantum dots (Figure 1), the transmission coefficient will have transmission resonances, transmission bands and transmission gaps. The shape of *T*(*E*) is sensitive to the geometry of the modulation and to the relative dimensions of the modulating parts of the wire. In Figure 2a, the transmission coefficient is shown for wire diameter modulation by one dot attached with two narrow constrictions. For the dimensions chosen here for illustration (Figure 1), the narrow constrictions have a propagation threshold of approximately 56 meV. Transmission resonances (R in Figure 2) occur in *T*(*E*) at electron energies at which no-propagating waves can exist in the narrow constrictions and which correspond to quasi-bound states of the dots. In this case, electronic transport is based on evanescent-mode coupling as in tunnelling phenomena in heterojunctions. Coupling between propagation resonances with small energy separation, result in the formation of narrow propagation bands (NB in Figure 2) in the transmission gap of the constrictions. Electron propagation states within the transmission gap of the constrictions can be interpreted as quasi-localized states.

**Figure 3:**
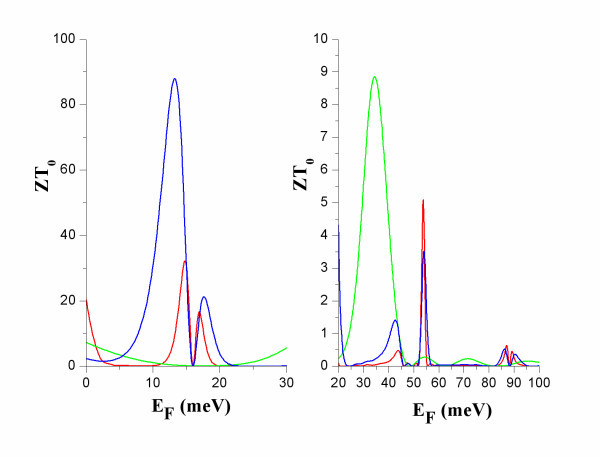
**The electron figure of merit *ZT***_**0 **_**versus *E***_**F **_**at *T *= 5 K (red), 10 K (blue) and 50 K (green)**.

At the transmission resonance R, *ZT*_0 _has been found to increase with increasing temperature *T *and decreasing broadening *Γ *(Figure 3). This behaviour can be qualitatively interpreted by the following expression that has been derived for an isolated resonance assuming a symmetric Lorentzian-function for the transmission coefficient [11]:(9)

where *Ε*_res _and *Γ *are the energy and the broadening of the resonance respectively.

**Figure 4:**
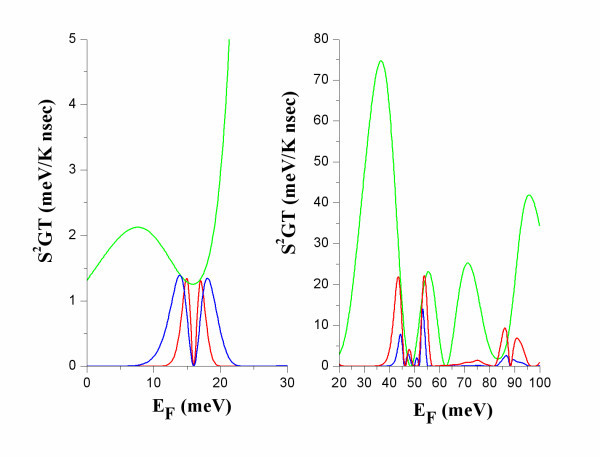
**The power factor *S***^**2**^***GT *versus *E***_**F **_**at *T *= 5 K (red), 10 K (blue) and 50 K (green)**.

At R, *ZT*_0 _(Figure 3) has very high values for small energy broadening *Γ *[9]. It should be noticed though, that the corresponding power factor is small (Figure 4). This can be interpreted by the following formula [11]:(10)

**Figure 5:**
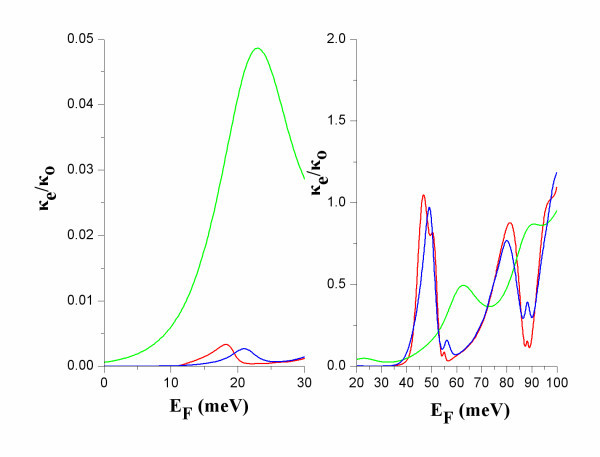
**The electron thermal conductance *κ*_*e *_versus *E*_F _at *T *= 5 K (red), 10 K (blue) and 50 K (green)**.

The transmission coefficient deviates from the symmetric-Lorentzian function because the transmission resonance R is not perfectly isolated. Due to coupling between R and the neighbouring transmission states, *T*(*E*) is asymmetric. At low temperatures, the asymmetry of *T*(*E*) has small effect on the conductance *G *and the thermopower *S*. *G *has a symmetric peak form, *S *is antisymmetric around the peak of *G *and *S*^2^*GT *has a double-peak form [2,4]. The asymmetry of *T*(*E*) has a more significant effect on the electron thermal conductance *κ*_e _that is also asymmetric with a peak shifted towards higher energies with respect to the zero of *S*^2^*GT *(Figure 5). *κ*_e _is sensitive to the shape of *T*(*E*) across the propagation resonance because the off-resonance states mainly contribute to it. The electron thermal conductance *κ*_e _would be zero for a single energy level with zero broadening (*Γ *= 0), as it has been shown for a single delta-like level of a quantum dot [2]. Due to the asymmetric *κ*_e_, *ZT*_0 _is also asymmetric (Figure 3). At elevated temperatures, Equation 9 is not a good approximation for *ZT*_0_, because then the electron distribution is thermally broadened and effects of coupling to neighbouring states become important.

At the narrow band below the transmission band threshold of the constriction, NB (Figure 2), *ZT*_0 _has considerably smaller values than at R due to higher electron thermal conductance at NB than at R (Figure 5). The power factor, *S*^2^*GT*, is considerably higher at NB than at R (Figure 4). At NB, a significant increase of *ZT*_0 _is found with increasing temperature due to: (i) increase of *S*^2^*GT *and (ii) decrease of *κ*_e _due to heat leakage through propagation band states at higher energies.

**Figure 6:**
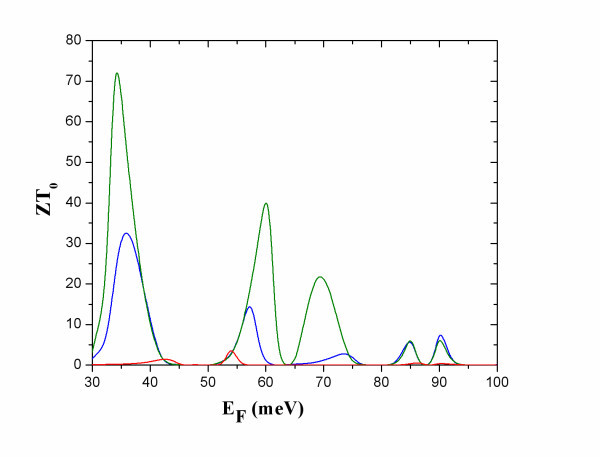
***ZT***_**0 **_**versus *E***_**F**_**, at *T *= 10 K, for nanowires modulated by one dot (red), three dots (blue) and five dots (dark green)**.

At NB, *ZT*_0 _increases when more modulating units are added (Figure 6) because a narrow band with sharper transmission thresholds is formed. This can be seen by comparing *T*(*E*) for a single dot and for a finite superlattice of three dots (Figure 2b). It should be noted that the formation of the transmission band edges is completed after a small number of periods and adding more dots does not result in any further increase in *ZT*_0_. It should, though, be emphasized that adding more modulating units could result in increase in *ZT *(Equation 7) due to decreased *κ*_ph_. Phonon conductance decreases due to additional phonon scattering when additional inhomogeneities are introduced in a nanowire.

It is well known that phonon conduction decreases in nanostructures due to phonon scattering on boundaries and interfaces. In heterostructures, optical and acoustic phonons have been shown to occupy quasi-bound states within narrow bands separated by gaps [12,13]. It has been shown [14,15] that in an ideal quantum wire the total acoustic phonon transmission probability exhibits perfect transmission steps. A perfect quantum thermal plateau exists, and at *T *= 0, its value approaches a universal value, . Quantum wires attached with inhomogeneities such as abrupt junctions and stub structures have already been reported [16-19]. In wires modulated by dots, the phonon transmission spectra have shown to display complex peak-dip structures. The thermal conductance plateau is destroyed and the thermal conductance decreases due to phonon scattering. It has been found [17-19] that the phonon thermal conductance can be adjusted by the attached scattering and it can become smaller than *κ*_0_. We have found that the electron thermal conductance can also be smaller than *κ*_0_. The ratio of the two contributions to the thermal conductance, *κ*_ph_/*κ*_e_, determines the value of coefficient *α*, i.e. the decrease of *ZT *relative to *ZT*_0_.

The low temperature phonon conductance of a wire modulated by dots has been estimated in Refs. [17-19]. We have taken into account the conclusions to estimate *ZT*.

**Figure 7:**
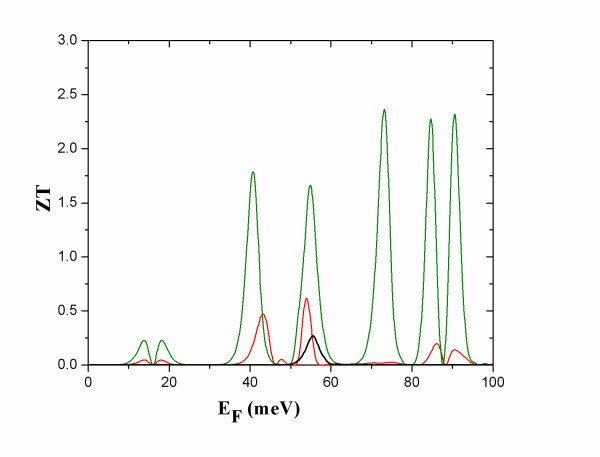
**The total figure of merit *ZT *versus *E***_**F**_. The curves are for : a uniform nanowire 10 nm thick (black) with *κ*_ph _= 1.5 *κ*_0_, and nanowires modulated by one dot (red) with *κ *_ph _= 0.5 *κ*_0 _and by five dots (dark green) with *κ *_ph _= 0.1 *κ*_0_**, at *T *= 10 K**.

At R, *ZT *is found much smaller than *ZT*_0 _(compare Figures 3 and 7). This is explained by that: (i) the power factor *S*^2^*GT *is small (Figure 4), and (ii) the reduction of *κ*_ph _is expected to be smaller than the reduction of *κ*_e_. In diameter modulated wires, wave interference effects result in reduction of the thermal conductance for both electrons and phonons. The significant reduction of *κ*_e _is due to the formation of transmission resonances for electrons and the energy selectivity provided by the Fermi distribution. Such transmission resonances have not been found for phonons [17-19]. More than one phonon modes contribute to the phonon conduction. Each mode has its own *T*(*E*) and energy selectivity is not provided by the phonon distribution. It is, therefore, unlike that, in diameter modulated nanowires, *κ*_ph _could be reduced as much as *κ*_e _at R. This remains, however, to be further explored.

At NB, *ZT *decreases relative to *ZT*_0 _but it has values close to and above 1 (Figure 7). This is because: (i) the power factor *S*^2^*GT *is not very small (Figure 4) in this case, and (ii) the ratio of *κ*_ph_/*κ*_e _can be of the order of 1. In the data shown in Figure 7, it has been considered that *κ*_ph _is always reduced due to the diameter modulation [17-19]. Hence, *ZT *of the modulated wire is found higher than that of a uniform thin wire with diameter equal to that of the thin constrictions (Figure 1). It is thereby indicated that increase of the thermoelectric efficiency of nanowires could be achieved by modulating their diameter instead of making them very thin and mechanically unstable.

In the ballistic regime, the thermoelectric efficiency of diameter modulated nanowires is directly related to the energy dependence of the transmission coefficient. For both electrons and phonons, *T*(*E*) is sensitive to the geometry of the modulated nanowires [9,17-19]. Geometry optimization of the diameter modulation could result in optimal electron thermoelectric properties and minimum phonon conduction. This task is very challenging because several geometry lengths are involved. For instance, the formation of quasi-localized states depends on the width and the length of the constrictions relative to the dimensions of the modulating units, by the number of modulating units, by disorder in arrays of non-identical modulation units. The recent progress in the fabrication of nanowires using well-controlled techniques (both epitaxial and etching) could allow for geometry optimization and development of efficient thermoelectric applications based on modulated nanowires.

## Conclusion

The thermoelectric efficiency of diameter modulated nanowires has been estimated by taking into account the electron and phonon transmission properties. It has been demonstrated that quasi-localized electron states can be formed that are prosperous for efficient thermoelectric energy conversion. Diameter modulated nanowires provide an architecture suitable for optimization of the transport properties of both electrons and phonons.

## Competing interests

The author declares that they have no competing interests.
